# Longitudinal association of exclusive and dual use of cigarettes and cigars with asthma exacerbation among US adults: a cohort study

**DOI:** 10.1186/s12931-024-02930-y

**Published:** 2024-08-10

**Authors:** Akash Patel, James H. Buszkiewicz, Steven Cook, Douglas A. Arenberg, Nancy L. Fleischer

**Affiliations:** 1 Center for Social Epidemiology and Population Health, Department of Epidemiology, School of Public Health, University of Michigan, Ann Arbor, MI 48109 USA; 2 Division of Pulmonary and Critical Medicine, Department of Internal Medicine, University of Michigan Medical School, Ann Arbor, MI 48109 USA

**Keywords:** Cigars, Cigarettes, Asthma exacerbation, Health effects

## Abstract

**Background:**

Cigar use among adults in the United States has remained relatively stable in the past decade and occupies a growing part of the tobacco marketplace as cigarette use has declined. While studies have established the detrimental respiratory health effects of cigarette use, the effects of cigar use need further characterization. In this study, we evaluate the prospective association between cigar use, with or without cigarettes, and asthma exacerbation.

**Methods:**

We used data from Waves 1–5 (2013–2019) of the Population Assessment of Tobacco and Health Study to run generalized estimating equation models examining the association between time-varying, one-wave-lagged cigarette and cigar use and self-reported asthma exacerbation among US adults (18+). We defined our exposure as non-established (reference), former, exclusive cigarette, exclusive cigar, and dual use. We defined an asthma exacerbation event as a reported asthma attack in the past 12 months necessitating oral or injected steroid medication or asthma symptoms disrupting sleep at least once a week in the past 30 days. We adjusted for age, sex, race and ethnicity, household income, health insurance, established electronic nicotine delivery systems use, cigarette pack-years, secondhand smoke exposure, obesity, and baseline asthma exacerbation.

**Results:**

Exclusive cigarette use (incidence rate ratio (IRR): 1.26, 95% confidence interval (CI): 1.03–1.54) and dual use (IRR: 1.41, 95% CI: 1.08–1.85) were associated with a higher rate of asthma exacerbation compared to non-established use, while former use (IRR: 1.01, 95% CI: 0.80–1.28) and exclusive cigar use (IRR: 0.70, 95% CI: 0.42–1.17) were not.

**Conclusion:**

We found no association between exclusive cigar use and self-reported asthma exacerbation. However, exclusive cigarette use and dual cigarette and cigar use were associated with higher incidence rates of self-reported asthma exacerbation compared to non-established use. Studies should evaluate strategies to improve cigarette and cigar smoking cessation among adults with asthma who continue to smoke.

**Supplementary Information:**

The online version contains supplementary material available at 10.1186/s12931-024-02930-y.

## Background

Asthma is a chronic respiratory disease characterized by inflammation and narrowing of the airways that impacted over 20 million adults in the United States (US) in 2021 [1, 2]. Clinical management of asthma has improved considerably over time, but many people continue to experience asthma exacerbations [3–6]. Asthma exacerbations are the worsening of asthma symptoms (e.g., increase in coughing or nocturnal coughing, wheezing, shortness of breath, chest tightness) and deterioration of lung function requiring a change in treatment [7, 8]. In 2021, nearly 40% of adults with asthma in the US experienced at least one asthma attack in the preceding 12 months [2]. Asthma exacerbations are associated with downstream health impacts, including decreased quality of life [9], decreased employment [10], and increased healthcare spending [11, 12]. Several individual-level (e.g., demographic, behavior, genetic, health), and environmental (e.g., allergens, infections, pollution) risk factors are associated with increased asthma exacerbations among adults, including combustible tobacco product use such as cigarettes [7, 8, 13–16].

From 2015 to 2021, prevalence of adults in the US who smoked cigarettes every day or some days decreased from 15.1% to 11.5% [17, 18]. However, cigarette use remains an important risk factor for asthma exacerbation. The 2014 Surgeon General’s report concluded, “The evidence is sufficient to infer a causal relationship between active smoking and exacerbation of asthma in adults.” [19] Studies have found an association between cigarette use and asthma exacerbations requiring healthcare visits (e.g. unscheduled physician visit, emergency department visit, hospitalization) [20–28], necessitating medication (e.g. corticosteroids) [22, 29, 30], or comprising of severe symptoms and frequent attacks [22, 31]. While the evidence showing the negative impact of cigarette use on asthma exacerbation is robust, less is known about the potential risks of asthma exacerbation attributable to cigar use or dual use of cigarettes and cigars.

Unlike cigarette use, prevalence of cigar use among adults in the US has remained relatively stable, hardly changing from 3.4% in 2015 to 3.5% in 2021 [17, 18]. A recent study found no association between cigar use and new onset or worsening asthma [32]. However, the association between cigar use and asthma or asthma exacerbation remains unclear; studies that examined these associations have limited sample size, inconsistent outcomes, and combined cigars with other tobacco products like pipes [33]. Still, adults who use cigars are exposed to nicotine and many of the same addictive, toxic, and carcinogenic constituents found in cigarettes [33, 34]. Even people who do not actively inhale cigar smoke are exposed to the high levels of environmental tobacco smoke that cigars produce [34]. Furthermore, cigarette and cigar use are intertwined; among adults who smoked non-premium cigars, more than half also smoked cigarettes [35]. Yet, we are unaware of any studies that have examined the association of dual use of cigarettes and cigars with asthma exacerbation.

Additional prospective studies and more clarity regarding the relationship between cigar use and asthma exacerbation are needed. In this study, we investigated the prospective association of self-reported asthma exacerbation by cigarette and cigar use status among a nationally representative sample of adults with asthma in the US.

## Methods

### Data

**Fig. 1:**
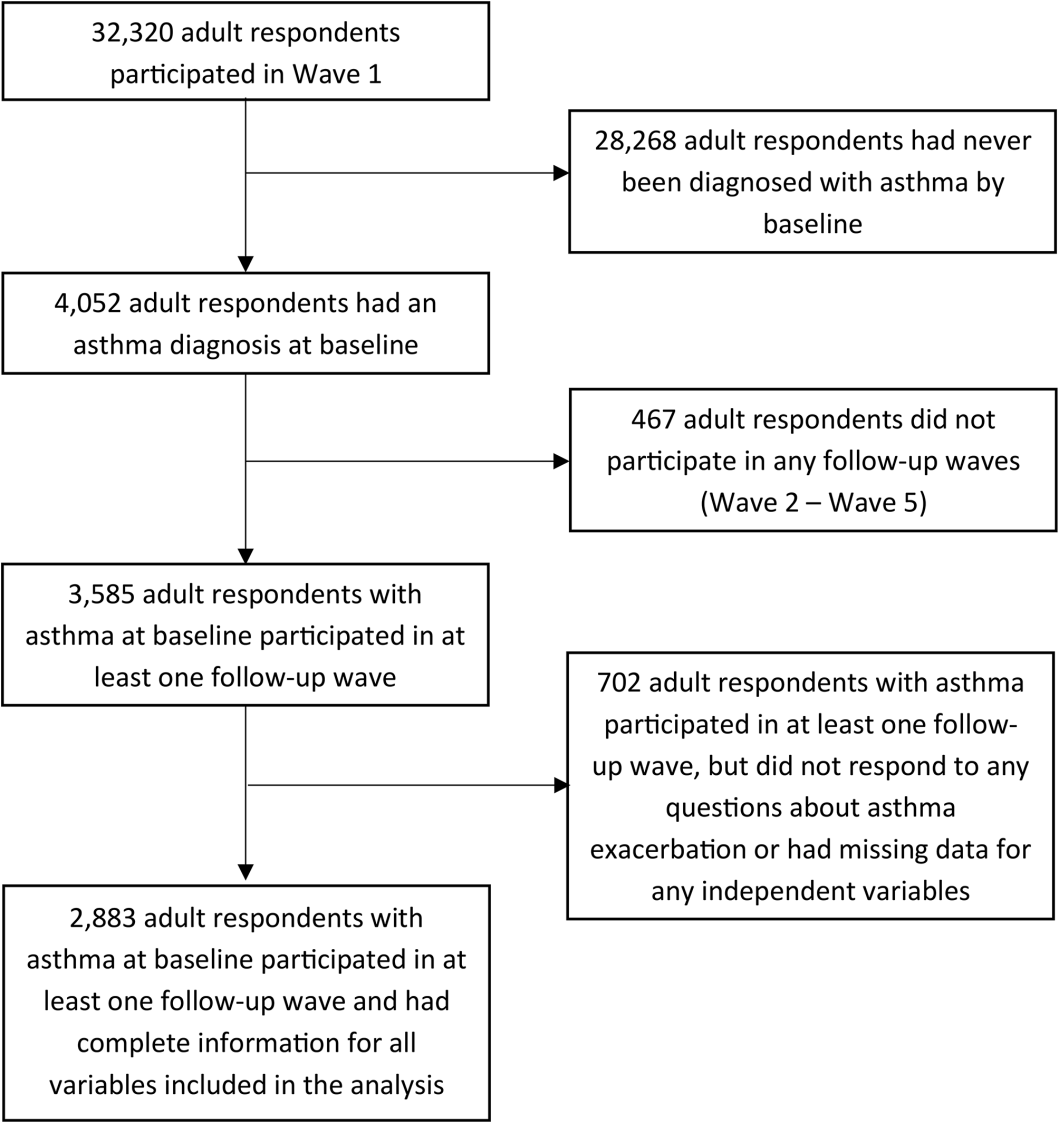
Selection of the analytic sample from the PATH Study

We used restricted adult (18+) data from Waves 1–5 (2013–2019) of the Population Assessment of Tobacco and Health (PATH) Study, an ongoing, nationally representative longitudinal cohort study of the civilian, non-institutionalized US population. Information about the PATH study design and how to access restricted PATH data files can be found elsewhere [36, 37]. In this study, our analytic sample consisted of 2,883 adult respondents previously diagnosed with asthma at baseline (Wave 1) who participated in at least one follow-up wave (Waves 2–5) and provided complete information related to the exposure, covariates, and outcome (Fig. 1). Our study was considered not regulated by the University of Michigan Institutional Review Board due to the use of secondary, de-identified data.

### Measure for asthma exacerbation

We used two questions to create a dichotomous variable indicating whether respondents experienced an asthma exacerbation at each follow-up wave. Respondents with an asthma diagnosis in Waves 3–5 were asked, “In the past 12 months, have you had an asthma attack that required the use of an oral or injected steroid medication at the time of the attack?” Notably, only respondents with asthma who also regularly used an oral or injected steroid medication in the past 12 months were asked the same question in Wave 2. From Waves 2–5, respondents with asthma were also asked, “In the past 30 days, how often did your asthma symptoms (such as wheezing, coughing, shortness of breath, chest tightness, or pain) wake you up at night or earlier than usual in the morning?” We considered respondents to have had an asthma exacerbation at each follow-up wave if they indicated using steroid medication for an asthma attack in the past 12 months or reported waking up at least one time per week in the past 30 days due to asthma symptoms.

### Measure for cigarettes and cigars use

We defined current cigarette use as having smoked cigarettes at least once in the past 30 days, established cigarette use as having smoked at least 100 cigarettes in a lifetime, and experimental cigarette use as having smoked cigarettes, but less than 100 in a lifetime. For cigars, we combined three cigar products – traditional cigars, cigarillos, and filtered cigars – and defined current cigar use as having used any cigar products at least once in the past 30 days, established cigar use as having used any cigar products fairly regularly, and experimental cigar use as having used any cigar product, but none fairly regularly.

Based on these definitions, we constructed an exposure variable at each wave with five categories: (1) never, former experimental, or experimental use [non-established cigarette and cigar use], (2) former established use of cigarettes or cigars [former cigarette or cigar use], (3) exclusive current established cigarette use [exclusive cigarette use], (4) exclusive current established cigar use [exclusive cigar use], and (5) current established dual use [dual use]. We then included this variable in our models as a time-varying exposure and lagged it by one wave to ensure the exposure temporally preceded the outcome. Our reference group was respondents who had never used cigarettes or cigars or had only experimented (formerly or currently) with either product.

### Covariates

We included age (continuous), sex (female, male), race and ethnicity (Hispanic, non-Hispanic (NH) White, NH Black, another NH race and ethnicity), annual household income ($50,000 or more, less than $50,000), and health insurance status (covered, not covered) as baseline sociodemographic covariates. We also adjusted for tobacco-related risk factors, including time-varying electronic nicotine delivery systems (ENDS) use and baseline cigarette pack-years. For time-varying ENDS use, we created a three-category (never or experimental use [non-established ENDS use], former established ENDS use [former ENDS use], current established ENDS use [current ENDS use]) variable, where ever fairly regular ENDS use was defined as established ENDS use and ENDS use at least once in the past 30 days was defined as current ENDS use. We calculated cigarette pack-years by multiplying the average number of packs-per-day and the years respondents smoked cigarettes to account for cigarette smoking history. Additionally, we controlled for other asthma exacerbation risk factors, including time-varying secondhand smoke exposure (number of 12-hour periods spent in close contact with others who were smoking in the past seven days), obesity (body mass index (BMI) ≥ 30.0) at baseline, and a prior asthma exacerbation at baseline (had an asthma attack in the past 12 months that required the use of an oral or injected steroid medication).

### Statistical analyses

We calculated weighted descriptive statistics to describe the overall analytic sample, respondents’ tobacco use behavior by cigarette and cigar use status at baseline, and the number of follow-up waves respondents experienced asthma exacerbations. Using an unbalanced person-period dataset, we ran generalized estimating equation (GEE) models to evaluate the prospective association between cigarette and cigar use and asthma exacerbation over three 1-year periods (Wave 1 – Wave 2, Wave 2 – Wave 3, Wave 3 – Wave 4) and one 2-year period (Wave 4 – Wave 5). For these models, we specified unstructured covariance, within-person correlation matrices, and a negative binomial distribution of the dependent variable using a log link function to estimate incidence rate ratios (IRR) and 95% confidence intervals (CI).

We excluded respondents who were missing exposure, covariate, or outcome information from the analytic sample and did not use any multiple imputation techniques. Literature examining complete case and multiple imputation analyses indicates that multiple imputation is more effective than complete case analysis when at least 5% to 10% of the data is missing [38, 39]. In our analysis, missingness ranged from 0.1% to 5.5% across covariates (Supplemental Table 1). Moreover, baseline characteristics of the analytic sample and the sample not excluding respondents with missing information were very similar, limiting concerns about missing data.

We weighted all data using Wave 1 weights to ensure that it was representative of the US civilian, non-institutionalized adult population at baseline, and we estimated variance using balanced repeated replication methods with Fay’s adjustment set to 0.3 [40]. We calculated descriptive statistics using Stata software, version 18.0 [41], and ran the weighted GEE analysis using modified code developed by Kasza et al. [42] with SAS V9.4 software [43].

### Sensitivity analyses

We ran multiple sensitivity analyses to confirm the robustness of our findings. First, we restricted the analysis to Waves 1–4 to examine how Wave 5 data impacted our results. Unlike earlier waves when PATH collected data annually, PATH began collecting data biennially with Wave 5, resulting in a two-year gap between Waves 4 and 5. Second, we re-ran the analysis using different weights to account for changes in non-response and eligibility across the waves. We used Wave 2 “single-wave” weights to restrict the analysis to respondents who participated in at least Waves 1 and 2 and Wave 5 “all-waves” weights to restrict the analysis to respondents who participated in all five follow-up waves. Third, we excluded respondents previously diagnosed with chronic obstructive pulmonary disease (COPD) at baseline following recommendations made in a study that examined the association between different tobacco product use and asthma [32]. We further replicated that study’s approach [32] and ran another analysis excluding respondents previously diagnosed with COPD, chronic bronchitis, emphysema, or some other lung or respiratory condition at baseline. Fourth, we excluded respondents from the analysis who exclusively smoked premium traditional cigars at baseline because they potentially misrepresented true established cigar use. A report on premium traditional cigars from the National Academies of Science, Engineering, and Medicine (NASEM) found differences in how premium traditional cigars are used compared to other cigar products [33]. People who smoked premium traditional cigars had lower frequency and intensity of smoking, were less likely to smoke cigarettes or other cigar products concurrently, and were more likely to be never or former cigarette smokers than those who used other cigar products [33]. Using previously described methods, we defined premium cigars based on brand name and price [33]. Fifth, we adjusted for other combustible tobacco products, hookah and pipe tobacco. For each product, we defined ever fairly regular use as established use and any past 30 day use as current use to create time-varying, three-category variables (never or experimental use, former established use, and current established use) indicating use status. Sixth, we adjusted for time-varying annual household income, time-varying health insurance status, and time-varying obesity rather than baseline values to capture potential changes over time. Lastly, we disaggregated the ‘non-established use’ category to create a seven-category exposure variable and changed the reference group to only respondents who had never used cigarettes or cigars.

## Results

### Sample demographics

At baseline, the mean age of our analytic sample of adults with an asthma diagnosis was 44.4 years (standard deviation (SD) = 17.4 years) (Table 1). They were predominantly female (61.7%), NH White (67.7%), had annual household incomes less than $50,000 (59.4%), and had health care coverage (90.0%). The average cigarette pack-years among sample respondents was 6.5 (SD = 15.1) and the average number of hours they were exposed to secondhand smoke in the past seven days was 7.3 h (SD = 21.6 h). Over 40% of respondents had a BMI greater than or equal to 30 kg/m^2^ (41.7%) and 15.4% had had an asthma exacerbation. Most respondents had non-established ENDS use, but 1.5% formerly used ENDS, and 2.4% currently used ENDS. Lastly, 60.5% of respondents had non-established cigarette or cigar use at baseline, 20.7% formerly smoked cigarettes or cigars, 16.5% exclusively used cigarettes, 1.0% exclusively used cigars, and 1.3% dual used cigarettes and cigars.

**Table 1:** Baseline characteristics of analytic sample, PATH Study (Wave 1, 2013-14)

	No. (%)^a^	95% CI
Time-varying cigarette and cigar use		
Non-established cigarette and cigar use	1334 (60.5)	58.1–62.8
Former cigarette or cigar use	481 (20.7)	18.6–23.0
Exclusive cigarette use	926 (16.5)	15.2–17.9
Exclusive cigar use	56 (1.0)	0.7–1.3
Dual use	86 (1.3)	1.1–1.7
*Sociodemographic risk factors*		
Age (mean (SD))	44.4 (17.4)	
Sex		
Female	1730 (61.7)	59.3–64.1
Male	1153 (38.3)	35.9–40.7
Race/Ethnicity		
Hispanic	461 (13.5)	11.9–15.3
NH White	1736 (67.7)	65.3–70.0
NH Black	441 (12.2)	10.9–13.6
Another NH race and ethnicity	245 (6.6)	5.4–8.1
Annual household income		
$50,000 or more	922 (40.6)	38.1–43.2
Less than $50,000	1961 (59.4)	56.8–61.9
Health insurance status		
Covered	2451 (90.0)	88.6–91.2
Not covered	432 (10.0)	8.8–11.4
*History of tobacco use*		
Time-varying ENDS use		
Non-established ENDS use	2653 (96.1)	95.5–96.7
Former ENDS use	92 (1.5)	1.2–1.8
Current ENDS use	138 (2.4)	1.9–2.9
Baseline cigarette pack-years (mean (SD))	6.5 (15.1)	
*Other risk factors*		
Time-varying secondhand smoking exposure - mean number of hours in past 7 days (mean (SD))	7.3 (21.6)	
Obesity (BMI ≥ 30.0) at baseline		
Yes	1120 (41.7)	39.0–44.4
No	1763 (58.3)	55.6–61.0
Asthma exacerbation at baseline		
Yes	407 (15.4)	13.5–17.6
No	2476 (84.6)	82.4–86.5

Abbreviations: PATH, Population Assessment of Tobacco and Health; CI, Confidence Interval; SD, Standard Deviation; NH, non-Hispanic; ENDS, Electronic Nicotine Delivery Systems; BMI, Body Mass Index

^a^ Unweighted counts and weighted percentages for categorical variables and weighted mean and standard deviation for continuous variables calculated using Wave 1 weights

### Tobacco use behavior

Established, current use of cigarettes and cigars remained consistent across Waves 1–4 (Supplemental Table 2). Generally, respondents who dual used cigarettes and cigars were more similar to those who exclusively smoked cigarettes than those who exclusively smoked cigars at baseline (Table 2). Specifically, both groups had a similar mean number of days they used cigarettes in the past 30 days (exclusive cigarettes: 26.8 days, dual use: 27.5 days), mean number of hours they were exposed to secondhand smoke in the past seven days (exclusive cigarettes: 22.5 h, dual use: 26.3 h), and cigarette smoking history (exclusive cigarettes: 16.2 pack-years, dual use: 15.8 pack-years). In contrast, respondents who exclusively smoked cigars smoked cigarettes experimentally an average of 7.8 days in the past 30 days, were exposed to secondhand smoke an average of 15.1 h in the past seven days, and averaged 4.0 pack-years of cigarette smoking. Respondents who exclusively used cigars or dual used cigarettes and cigars both used filtered cigars an average of 5.6 days in the past 30 days, but there were slight differences in the average number of days these respondents used cigarillos (exclusive cigars: 6.4 days, dual use: 5.2 days) and traditional cigars (exclusive cigars: 3.2 days, dual use: 4.2 days) in the past 30 days. Lastly, respondents who formerly smoked cigarettes or cigars had the highest number of cigarette pack-years (17.3 pack-years) but were otherwise similar to respondents with non-established cigarette or cigar use.

**Table 2:** Baseline tobacco use characteristics by cigarette and cigar use behavior, PATH Study (Wave 1, 2013-14)

	Non-established cigarette and cigar use (*n* = 1,334)		Former cigarette or cigar use (*n* = 481)		Exclusive cigarette use (*n* = 926)		Exclusive cigar use (*n* = 56)		Dual use (*n* = 86)	
	No. (%)^a^	95% CI	No. (%)^a^	95% CI	No. (%)^a^	95% CI	No. (%)^a^	95% CI	No. (%)^a^	95% CI
*Current (1 + days in past 30 days) established tobacco use*										
Cigarettes					926 (100)				86 (100)	
Cigar Type										
Traditional cigars							22 (46.5)	32.5–61.1	36 (41.9)	31.5–53.1
Filtered cigars							11 (21.0)	11.2–35.9	23 (27.8)	18.7–39.1
Cigarillos							31 (45.8)	32.4–59.7	45 (50.4)	39.2–61.6
Poly cigars							8 (13.3)	6.4–25.7	18 (20.1)	12.3–31.1
*Mean number of days used in the past 30 days*										
Cigarettes (mean (SD))	0.6 (3.5)		0.3 (2.8)		26.8 (10.7)		7.8 (17.5)		27.5 (9.7)	
Traditional cigars (mean (SD))	0.0 (0.4)		0.0 (0.4)		0.1 (2.1)		3.2 (9.5)		4.2 (12.3)	
Filtered cigars (mean (SD))	0.0 (0.2)		0.0 (0.1)		0.0 (0.6)		5.6 (16.0)		5.6 (16.0)	
Cigarillos (mean (SD))	0.0 (0.7)		0.0 (0.1)		0.3 (3.4)		6.4 (14.3)		5.2 (13.6)	
Time-varying secondhand smoking exposure - mean number of hours of exposure in past 7 days (mean (SD))	3.6 (13.0)		4.5 (16.1)		22.5 (47.0)		15.1 (49.1)		26.3 (48.9)	
Cigarette pack-years (mean (SD))	0.0 (0.0)		17.3 (19.9)		16.2 (25.0)		4.0 (18.6)		15.8 (31.1)	

Abbreviations: PATH, Population Assessment of Tobacco and Health; CI, Confidence Interval; SD, Standard Deviation; ENDS, Electronic Nicotine Delivery Systems

^a^ Unweighted counts and weighted percentages for categorical variables and weighted mean and standard deviation for continuous variables calculated using Wave 1 weights

### Asthma exacerbation events

In the four follow-up waves examined, most respondents (64.2%) did not experience an asthma exacerbation event (Table 3). Nearly half of all respondents who experienced an asthma exacerbation event in at least one wave (*n* = 1,018) had an asthma exacerbation event in two or more waves. Overall, 18.7% had an asthma exacerbation event in one wave, 8.3% in two, 5.8% in three, and 3.0% in four waves.

**Table 3:** Total number of waves experienced asthma exacerbations, PATH Study (Waves 2–5, 2013-19)

Number of follow-up waves	No. (%)^a^ (*N* = 2,883)	95% CI
0	1865 (64.2)	61.8–66.6
1	519 (18.7)	16.7–20.9
2	249 (8.3)	7.1–9.6
3	159 (5.8)	4.8–7.0
4	91 (3.0)	2.2–4.0

Abbreviations: PATH, Population Assessment of Tobacco and Health; CI, Confidence Interval

^a^ Unweighted counts and weighted percentages calculated using Wave 1 weights

### Association between cigarette and cigar use and asthma exacerbation

In the unadjusted GEE model, respondents who exclusively smoked cigarettes (IRR: 1.52, 95% CI: 1.26–1.82) or dual used cigarettes and cigars (IRR: 1.72, 95% CI: 1.31–2.24) had higher rates of asthma exacerbation compared to those who had non-established cigarette and cigar use (Table 4, Model 1). The rate of asthma exacerbation for respondents who formerly smoked cigarettes or cigars (IRR: 1.21, 95% CI: 1.00–1.46) or exclusively smoked cigars (IRR: 0.73, 95% CI: 0.44–1.20) did not differ statistically from the rate for respondents with non-established use. Adjusting for baseline sociodemographic, tobacco use history, and other asthma exacerbation risk factors (Table 4, Model 4) attenuated the estimated IRRs but did not change the statistical significance of the findings. Exclusive cigarette use (IRR: 1.26, 95% CI: 1.03–1.54) and dual use (IRR: 1.41, 95% CI: 1.08–1.85) had higher rates of asthma exacerbation, whereas former use of either product (IRR: 1.01, 95% CI: 0.80–1.28) and exclusive cigar use (IRR: 0.70, 95% CI: 0.42–1.17) did not differ statistically from non-established use of either product. Also of note, in the final adjusted model, respondents with current ENDS use had higher rates of asthma exacerbation (IRR: 1.20, 95% CI: 1.02–1.40) than respondents who had non-established ENDS use.

**Table 4:** GEE models predicting incidence rate ratio of asthma exacerbation, PATH Study (Waves 1–5, 2013-19)^a^

	Model 1^b^		Model 2^c^		Model 3^d^		Model 4^e^	
	IRR (95% CI)	*p*-value	IRR (95% CI)	*p*-value	IRR (95% CI)	*p*-value	IRR (95% CI)	*p*-value
Period								
1 (Wave 1 – Wave 2)	Reference		Reference		Reference		Reference	
2 (Wave 2 – Wave 3)	1.38 (1.18–1.60)	0.00006	1.38 (1.19–1.60)	0.00004	1.38 (1.19–1.60)	0.00004	1.38 (1.19–1.60)	0.00005
3 (Wave 3 – Wave 4)	1.38 (1.18–1.60)	0.00006	1.38 (1.19–1.61)	0.00004	1.38 (1.19–1.61)	0.00004	1.38 (1.19–1.61)	0.00005
4 (Wave 4 – Wave 5)	1.15 (0.99–1.34)	0.07612	1.16 (0.99–1.35)	0.06650	1.15 (0.99–1.35)	0.07271	1.16 (0.99–1.36)	0.06575
Time-varying cigarette and cigar use								
Non-established cigarette and cigar use	Reference		Reference		Reference		Reference	
Former cigarette or cigar use	1.21 (1.00–1.46)	0.05240	1.09 (0.89–1.32)	0.40649	1.00 (0.79–1.27)	0.97864	1.01 (0.80–1.28)	0.91494
Exclusive cigarette use	1.52 (1.26–1.82)	0.00002	1.44 (1.21–1.71)	0.00007	1.29 (1.05–1.59)	0.01385	1.26 (1.03–1.54)	0.02694
Exclusive cigar use	0.73 (0.44–1.20)	0.20933	0.76 (0.45–1.27)	0.29535	0.71 (0.42–1.20)	0.19283	0.70 (0.42–1.17)	0.17201
Dual use	1.72 (1.31–2.24)	0.00012	1.70 (1.34–2.15)	0.00003	1.50 (1.15–1.98)	0.00366	1.41 (1.08–1.85)	0.01283
*Sociodemographic risk factors*								
Age			1.02 (1.01–1.02)	0.00000	1.02 (1.01–1.02)	0.00000	1.02 (1.01–1.02)	0.00000
Sex								
Female			1.29 (1.08–1.53)	0.00488	1.29 (1.08–1.53)	0.00488	1.19 (1.00–1.41)	0.04839
Male			Reference		Reference		Reference	
Race/Ethnicity								
Hispanic			1.21 (0.98–1.49)	0.07740	1.24 (1.00–1.53)	0.04978	1.17 (0.91–1.49)	0.22339
NH White			Reference		Reference		Reference	
NH Black			1.02 (0.82–1.27)	0.84513	1.04 (0.84–1.30)	0.69351	1.00 (0.80–1.24)	0.96988
Another NH race and ethnicity			1.15 (0.76–1.74)	0.50324	1.16 (0.77–1.74)	0.48523	1.21 (0.81–1.79)	0.35106
Annual household income								
$50,000 or more			0.59 (0.47–0.75)	0.00004	0.60 (0.47–0.77)	0.00006	0.62 (0.49–0.78)	0.00005
Less than $50,000			Reference		Reference		Reference	
Health insurance status								
Covered			0.83 (0.66–1.05)	0.11990	0.84 (0.67–1.05)	0.12335	0.82 (0.66–1.03)	0.08357
Not covered			Reference		Reference		Reference	
*History of tobacco use*								
Time-varying ENDS use								
Non-established ENDS use					Reference		Reference	
Former ENDS use					1.11 (0.94–1.31)	0.20131	1.05 (0.90–1.23)	0.49642
Current ENDS use					1.25 (1.08–1.46)	0.00342	1.20 (1.02–1.40)	0.02395
Log of baseline cigarette pack-years / 10					1.11 (0.95–1.28)	0.17673	1.13 (0.97–1.31)	0.10428
*Other risk factors*								
Time-varying secondhand smoking exposure - mean number of 12 h of exposure in past 7 days							1.02 (1.00–1.05)	0.07777
Obesity (BMI ≥ 30.0) at baseline								
Yes							1.11 (0.94–1.30)	0.20851
No							Reference	
Asthma exacerbation at baseline								
Yes							2.39 (2.09–2.74)	0.00000
No							Reference	

Abbreviations: GEE, Generalized Estimating Equation; PATH, Population Assessment of Tobacco and Health; IRR, Incidence Rate Ratio; CI, Confidence Interval; NH, non-Hispanic; ENDS, Electronic Nicotine Delivery Systems; BMI, Body Mass Index

^a^ Total number of participants = 2883, Total number of observations = 9514

^b^ Adjusting for cigarette and cigar use only using Wave 1 weights

^c^ Adjusting for cigarette and cigar use and sociodemographics using Wave 1 weights

^d^ Adjusting for cigarette and cigar use, sociodemographics, and tobacco use history using Wave 1 weights

^e^ Adjusting for cigarette and cigar use, sociodemographics, tobacco use history, and other risk factors using Wave 1 weights

### Sensitivity analyses

The results of the sensitivity analyses were consistent with our main findings (Supplemental Tables 3–11). Notably, the association found between current ENDS use and asthma exacerbation was no longer statistically significant except in the sensitivity analyses using Wave 2 “single-wave” weights (Supplemental Table 4), adjusting for hookah and pipe tobacco use (Supplemental Table 9), adjusting for time-varying annual household income, health insurance status, and obesity (Supplemental Table 10), and using the exposure variable disaggregating non-established use (Supplemental Table 11).

## Discussion

We examined the prospective association of cigarette and cigar use with asthma exacerbation incidence among US adults using nationally representative data from PATH Waves 1–5 (2013–2019) and adjusting for baseline sociodemographic, tobacco use history, and other asthma exacerbation risk factors. We found that exclusive cigarette use and dual use of cigarettes and cigars were both associated with higher rates of asthma exacerbation compared to non-established use of either product. We consistently detected these associations across a series of sensitivity analyses. However, we found no statistically significant association for former cigarette or cigar use, nor for exclusive cigar use in the five-year follow-up period.

Our findings regarding the impact of cigarette use on asthma exacerbation align with those from past studies that found an association between current cigarette use and higher rates of asthma exacerbation [19]. A recent study that assessed the influence of active tobacco smoking on asthma outcomes found asthmatic patients who currently smoked cigarettes had twice the mean number of asthma exacerbations per year as patients who never smoked cigarettes and patients who formerly smoked cigarettes [30]. Adults with asthma who smoke cigarettes have a greater decline in lung function, worse asthma severity, less control over asthma symptoms, increased rates of hospitalization, attenuated response to corticosteroids, greater need for rescue medications, and higher morbidity and mortality rates than those with asthma who do not smoke cigarettes [44, 45]. However, prevalence of cigarette use among adults with asthma is similar to the general population [45, 46]. We found no association between former cigarette or cigar use and asthma exacerbation incidence, suggesting that smoking cessation may be a necessary and effective method to decrease asthma exacerbations.

Our study adds to the growing literature examining the health impacts of cigar use and dual use with cigarettes. Cigar product use is heterogeneous, ranging from premium traditional cigars that are smoked occasionally to filtered cigars that are smoked more frequently [35]. Adding further complexity, cigar use appears to be more unstable than cigarette use and how cigar products are used depends on past cigarette use [33, 47]. We found no association between exclusive cigar use and asthma exacerbation, but dual use of cigarettes and cigars was associated with higher rates of asthma exacerbation compared to non-established use. This is consistent with our observation that adults with asthma who used both products were more similar to adults who exclusively smoked cigarettes than those who exclusively smoked cigars in terms of tobacco use behavior and history at baseline. Our findings further suggest that adults who dual use may have higher rates of asthma exacerbation incidence than adults who exclusively use cigarettes. Inhalation of cigar smoke, known to be associated with adverse health effects [33, 34], is a likely explanation if these findings can be confirmed. Prior research has found that adults who smoke cigars are more likely to inhale if they ever smoked cigarettes than if they never smoked cigarettes [33, 34]. Furthermore, people who dual use cigarettes and cigars are more likely to smoke cigars with greater intensity and inhale smoke more intensely than people who exclusively smoke cigars [33, 34].

In our final model, we also adjusted for ENDS use. Prevalence of e-cigarette use among US adults has steadily increased from 3.5% in 2015 to 4.5% in 2021 [17, 18]. Multiple studies examining the acute respiratory effects of ENDS use found that short-term ENDS use decreased pulmonary functions and increased airway inflammation among individuals with asthma [48–51], but at least one study did not [52]. We found that current ENDS use was associated with higher rates of asthma exacerbation compared to non-established ENDS use. However, in several sensitivity analyses, this association was no longer statistically significant. The evidence for an association between ENDS use and asthma exacerbation in the literature is inconclusive [53], with some studies finding an association [54–56] and others not [32, 57]. Further research is needed before any conclusions can be drawn because data examining the long-term health effects of ENDS use are limited [53]. As more data becomes available and items measuring ENDS use are standardized, studies should also consider assessing what impact, if any, ENDS initiation, duration of use, frequency or intensity of use, device type, and flavoring have on asthma and other respiratory disease outcomes. For now, even if ENDS are safer alternatives to combustible tobacco products, researchers and clinicians should be cautious when advocating for ENDS use as a smoking cessation method for adults with asthma. ENDS aerosols are still poorly characterized, long-term health impacts are unknown, and airway alterations may place adults with asthma at higher risk for complications.

Our study has several strengths. First, we build upon growing literature examining the impact of multiple tobacco product use on asthma exacerbation [32, 56]. Second, we used prospective, nationally representative data from the PATH Study increasing the generalizability of our findings. Third, we corroborated our findings by conducting rigorous sensitivity analyses that considered multiple weighting schemes and additional adjustments. Finally, to the best of our knowledge, this is the only study to date that has prospectively evaluated the impact of dual cigarette and cigar use on asthma exacerbation.

However, some limitations of this study need to be acknowledged. First, we used self-reported measures which are subject to response biases. Social desirability may also have led to underreporting of tobacco use due to potential stigma against adults with asthma who continue to use combustible tobacco products. Still, this would have biased our results towards the null and supported the robustness of our findings. Second, we aggregated traditional cigars, cigarillos, and filtered cigars due to sample size concerns (*n* = 56 for exclusive current established any cigar use at baseline) and were therefore unable to examine asthma exacerbation risk associated with individual cigar product types alone or combined with cigarettes, which may be important given the variation in both the frequency and intensity of use across product types. Future studies should recruit larger samples of adults who smoke cigars to garner sufficient power needed to evaluate the risk posed by different cigar types. Third, our analysis was limited to the data that was collected in the PATH Study. We were unable to adjust for important asthma exacerbation risk factors, such as allergies or atopies, viral upper respiratory tract infections, and environmental air pollution [7, 13, 14], which may have confounded our analysis. Furthermore, we were unable to account for the level of cigar smoke inhalation and we could not examine the frequency and severity of asthma exacerbations.

## Conclusion

Findings from this study suggest that time-varying exclusive cigarette use and dual use of cigarettes and cigars were associated with higher asthma exacerbation rates compared to non-established use of cigarettes and cigars among US adults with asthma using nationally representative data. Neither time-varying former cigarette or cigar use, nor exclusive cigar use were statistically associated with asthma exacerbation. There is a need to develop more effective smoking cessation interventions for adults with asthma who continue using combustible tobacco products, especially those who dual use. Additionally, as the tobacco marketplace changes and new policies are implemented, further studies will be needed to better understand how tobacco products like cigars and ENDS are associated with asthma exacerbation risk and their impact on the frequency and severity of asthma exacerbations.

### Electronic supplementary material

Below is the link to the electronic supplementary material.

Supplementary Material 1

## Data Availability

The dataset generated and analyzed in the current study used restricted use data from the PATH Study because the analysis included cigarette pack-years and continuous age. Details on how to access restricted use data from the PATH Study is described in the PATH Study Restricted Use Files User Guide: https://doi.org/10.3886/ICPSR36231.v37. Researchers interested in obtaining these data from the Inter-university Consortium for Political and Social Research (ICPSR) at the University of Michigan must complete a Restricted Data Use Agreement. For additional information, please reference: https://www.icpsr.umich.edu/web/pages/NAHDAP/vde/index.html.

## Declarations

### Disclaimer

The opinions expressed in this work are the authors’ own and do not reflect the views of the National Institutes of Health, the Food and Drug Administration, the Department of Health and Human Services, nor the United States government.

## Abbreviations

US: United States
PATH: Population Assessment of Tobacco and Health
NH: Non–Hispanic
ENDS: Electronic Nicotine Delivery Systems
BMI: Body Mass Index
GEE: Generalized Estimating Equation
IRR: Incidence Rate Ratio
CI: Confidence Interval
COPD: Chronic Obstructive Pulmonary Disease
NASEM: National Academies of Science, Engineering, and Medicine
SD: Standard Deviation
