# Supplementary material for: Longitudinal association of exclusive and dual use of cigarettes and cigars with asthma exacerbation among US adults: a cohort study
Source: Respir Res. 2024 Aug 10;25:305. doi: 10.1186/s12931-024-02930-y (PMC11316995; doi:10.1186/s12931-024-02930-y)
Supplement: Supplementary file 1 — Supplementary Material 1 [file 12931_2024_2930_MOESM1_ESM.docx]

**Supplemental Material**

| **Table S1.** Baseline characteristics of samples excluding and not excluding respondents missing exposure, covariate, and outcome information, PATH Study (Wave 1, 2013-14)^a^ | | | | | | |
| --- | --- | --- | --- | --- | --- | --- |
|  | Sample excluding respondents missing exposure, covariate, and outcome information (N = 2,883) | | | Sample not excluding respondents missing exposure, covariate, and outcome information (N = 3,585) | | |
|  | No. (%)^b^ | 95% CI | Mean (SD)^c^ | No. (%)^b^ | 95% CI | Mean (SD)^c^ |
| Baseline cigarette and cigar use |  |  |  |  |  |  |
| Non-established cigarette and cigar use | 1334 (60.5) | 58.1 - 62.8 |  | 1602 (58.1) | 55.8 - 60.4 |  |
| Former cigarette or cigar use | 481 (20.7) | 18.6 - 23.0 |  | 619 (21.6) | 19.6 - 23.7 |  |
| Exclusive cigarette use | 926 (16.5) | 15.2 - 17.9 |  | 1158 (17.0) | 15.9 - 18.3 |  |
| Exclusive cigar use | 56 (1.0) | 0.7 - 1.3 |  | 74 (1.0) | 0.8 - 1.3 |  |
| Dual use | 86 (1.3) | 1.1 - 1.7 |  | 101 (1.3) | 1.1 - 1.7 |  |
| Missing |  |  |  | 31 (0.9) | 0.5 - 1.7 |  |
| *Sociodemographic risk factors* |  |  |  |  |  |  |
| Sex |  |  |  |  |  |  |
| Female | 1730 (61.7) | 59.3 - 64.1 |  | 2126 (61.4) | 59.2 - 63.5 |  |
| Male | 1153 (38.3) | 35.9 - 40.7 |  | 1455 (38.4) | 36.3 - 40.6 |  |
| Missing |  |  |  | 4 (0.2) | 0.0 - 0.6 |  |
| Race/Ethnicity |  |  |  |  |  |  |
| Hispanic | 461 (13.5) | 11.9 - 15.3 |  | 574 (13.6) | 12.1 - 15.4 |  |
| NH White | 1736 (67.7) | 65.3 - 70.0 |  | 2102 (65.4) | 63.1 - 67.7 |  |
| NH Black | 441 (12.2) | 10.9 - 13.6 |  | 533 (11.7) | 10.6 - 13.0 |  |
| Another NH race and ethnicity | 245 (6.6) | 5.4 - 8.1 |  | 308 (7.0) | 5.6 - 8.6 |  |
| Missing |  |  |  | 68 (2.3) | 1.6 - 3.3 |  |
| Annual household income |  |  |  |  |  |  |
| $50,000 or more | 922 (40.6) | 38.1 - 43.2 |  | 1052 (37.2) | 34.9 - 39.7 |  |
| Less than $50,000 | 1961 (59.4) | 56.8 - 61.9 |  | 2341 (57.2) | 54.7 - 59.7 |  |
| Missing |  |  |  | 192 (5.5) | 4.5 - 6.8 |  |
| Health insurance status |  |  |  |  |  |  |
| Covered | 2451 (90.0) | 88.6 - 91.2 |  | 3008 (88.6) | 87.3 - 89.8 |  |
| Not covered | 432 (10.0) | 8.8 - 11.4 |  | 550 (10.5) | 9.4 - 11.7 |  |
| Missing |  |  |  | 27 (0.8) | 0.5 - 1.5 |  |
| *History of tobacco use* |  |  |  |  |  |  |
| Baseline cigarette pack-years |  |  |  |  |  |  |
| Non-missing | 2883 (100.0) |  | 6.5 (15.1) | 3492 (97.0) | 96.1 - 97.8 | 6.6 (15.1) |
| Missing |  |  |  | 93 (3.0) | 2.2 - 3.9 |  |
| *Other risk factors* |  |  |  |  |  |  |
| Baseline secondhand smoking exposure - mean number of hours in past 7 days |  |  |  |  |  |  |
| Non-missing | 2883 (100.0) |  | 7.3 (21.6) | 3553 (99.5) | 99.3 - 99.7 | 7.2 (21.1) |
| Missing |  |  |  | 32 (0.5) | 0.3 - 0.7 |  |
| Obesity (BMI ≥ 30.0) at baseline |  |  |  |  |  |  |
| Yes | 1120 (41.7) | 39.0 - 44.4 |  | 1348 (39.7) | 37.3 - 42.2 |  |
| No | 1763 (58.3) | 55.6 - 61.0 |  | 2183 (58.2) | 55.8 - 60.6 |  |
| Missing |  |  |  | 54 (2.0) | 1.3 - 3.0 |  |
| Asthma exacerbation at baseline |  |  |  |  |  |  |
| Yes | 407 (15.4) | 13.5 - 17.6 |  | 492 (14.8) | 13.2 - 16.6 |  |
| No | 2476 (84.6) | 82.4 - 86.5 |  | 3087 (85.0) | 83.2 - 86.7 |  |
| Missing |  |  |  | 6 (0.1) | 0.1 - 0.3 |  |
| Abbreviations: PATH, Population Assessment of Tobacco and Health; SD, Standard Deviation; CI, Confidence Interval; NH, non-Hispanic; BMI, Body Mass Index | | | | | | |
| ^a^ Age and time-varying ENDS use not included in the table as they did not meet the minimum threshold for disclosure. | | | | | | |
| ^b^ Unweighted counts and weighted percentages calculated using Wave 1 weights | | | | | | |
| ^c^ Weighted mean and standard deviation calculated for continuous variables (non-missing only) using Wave 1 weights | | | | | | |

| **Table S2.** Cigarette and cigar use behavior over time, PATH Study (Wave 1-4, 2013-17) | | | | | | | | |
| --- | --- | --- | --- | --- | --- | --- | --- | --- |
| Time-varying cigarette and cigar use | Wave 1 (n = 2,883) | | Wave 2 (n = 2,432) | | Wave 3 (n = 2,237) | | Wave 4 (n = 1,962) | |
|  | No. (%)^a^ | 95% CI | No. (%)^a^ | 95% CI | No. (%)^a^ | 95% CI | No. (%)^a^ | 95% CI |
| Non-established cigarette and cigar use | 1334 (60.5) | 58.1 - 62.8 | 1027 (57.2) | 54.5 - 59.9 | 910 (56.0) | 53.1 - 59.0 | 776 (55.5) | 52.4 - 58.4 |
| Former cigarette or cigar use | 481 (20.7) | 18.6 - 23.0 | 504 (23.8) | 21.3 - 26.4 | 515 (25.1) | 22.5 - 27.8 | 481 (25.4) | 22.8 - 28.2 |
| Exclusive cigarette use | 926 (16.5) | 15.2 - 17.9 | 784 (16.5) | 15.0 - 18.2 | 710 (16.6) | 14.9 - 18.4 | 601 (16.2) | 14.5 - 18.1 |
| Exclusive cigar use | 56 (1.0) | 0.7 - 1.3 | 45 (0.9) | 0.7 - 1.3 | 37 (0.9) | 0.6 - 1.3 | 39 (1.2) | 0.8 - 1.6 |
| Dual use | 86 (1.3) | 1.1 - 1.7 | 72 (1.6) | 1.1 - 2.3 | 65 (1.4) | 1.1 - 1.9 | 65 (1.8) | 1.3 - 2.5 |
| Abbreviations: PATH, Population Assessment of Tobacco and Health; CI, Confidence Interval | | | | | | | | |
| ^a^ Unweighted counts and weighted percentages calculated using Wave 1 weights | | | | | | | | |

| **Table S3**. GEE model predicting incidence rate ratio of asthma exacerbation excluding Wave 5, PATH Study (Wave 1-4, 2013-17)^a,b^ | | |
| --- | --- | --- |
|  | IRR (95% CI) | p-value |
| Period |  |  |
| 1 (Wave 1 – Wave 2) | Reference |  |
| 2 (Wave 2 – Wave 3) | 1.38 (1.18 - 1.60) | 0.00005 |
| 3 (Wave 3 – Wave 4) | 1.38 (1.19 - 1.60) | 0.00006 |
| Time-varying cigarette and cigar use |  |  |
| Non-established cigarette and cigar use | Reference |  |
| Former cigarette or cigar use | 1.06 (0.84 - 1.34) | 0.61378 |
| Exclusive cigarette use | 1.29 (1.05 - 1.58) | 0.01399 |
| Exclusive cigar use | 0.75 (0.43 - 1.32) | 0.31921 |
| Dual use | 1.47 (1.11 - 1.96) | 0.00785 |
| *Sociodemographic risk factors* |  |  |
| Age | 1.02 (1.01 - 1.02) | 0.00000 |
| Sex |  |  |
| Female | 1.18 (0.99 - 1.40) | 0.06646 |
| Male | Reference |  |
| Race/Ethnicity |  |  |
| Hispanic | 1.13 (0.88 - 1.45) | 0.34189 |
| NH White | Reference |  |
| NH Black | 0.95 (0.74 - 1.23) | 0.70902 |
| Another NH race and ethnicity | 1.24 (0.82 - 1.86) | 0.30324 |
| Annual household income |  |  |
| $50,000 or more | 0.64 (0.51 - 0.81) | 0.00022 |
| Less than $50,000 | Reference |  |
| Health insurance status |  |  |
| Covered | 0.80 (0.63 - 1.00) | 0.05392 |
| Not covered | Reference |  |
| *History of tobacco use* |  |  |
| Time-varying ENDS use |  |  |
| Non-established ENDS use | Reference |  |
| Former ENDS use | 1.04 (0.88 - 1.23) | 0.66072 |
| Current ENDS use | 1.13 (0.94 - 1.35) | 0.19682 |
| Log of baseline cigarette pack-years / 10 | 1.09 (0.93 - 1.29) | 0.28812 |
| *Other risk factors* |  |  |
| Time-varying secondhand smoking exposure - mean number of 12 hours of exposure in past 7 day | 1.02 (1.00 - 1.05) | 0.04878 |
| Obesity (BMI ≥ 30.0) at baseline |  |  |
| Yes | 1.14 (0.97 - 1.35) | 0.11420 |
| No | Reference |  |
| Asthma exacerbation at baseline |  |  |
| Yes | 2.42 (2.09 - 2.82) | 0.00000 |
| No | Reference |  |
| Abbreviations: GEE, Generalized Estimating Equation; PATH, Population Assessment of Tobacco and Health; IRR, Incidence Rate Ratio; CI, Confidence Interval; NH, non-Hispanic; ENDS, Electronic Nicotine Delivery Systems; BMI, Body Mass Index | | |
| ^a^ Total number of participants = 2883, Total number of observations = 7552 | | |
| ^b^ Adjusting for cigarette and cigar use, sociodemographics, tobacco use history, and other risk factors using Wave 1 weights | | |

| **Table S4**. GEE model predicting incidence rate ratio of asthma exacerbation using Wave 2 weights, PATH Study (Wave 1-5, 2013-19)^a,b^ | | |
| --- | --- | --- |
|  | IRR (95% CI) | p-value |
| Period |  |  |
| 1 (Wave 1 – Wave 2) | Reference |  |
| 2 (Wave 2 – Wave 3) | 1.38 (1.18 - 1.61) | 0.00009 |
| 3 (Wave 3 – Wave 4) | 1.38 (1.19 - 1.61) | 0.00006 |
| 4 (Wave 4 – Wave 5) | 1.16 (0.98 - 1.36) | 0.08543 |
| Time-varying cigarette and cigar use |  |  |
| Non-established cigarette and cigar use | Reference |  |
| Former cigarette or cigar use | 1.02 (0.81 - 1.29) | 0.85064 |
| Exclusive cigarette use | 1.27 (1.03 - 1.55) | 0.02243 |
| Exclusive cigar use | 0.66 (0.39 - 1.11) | 0.11586 |
| Dual use | 1.44 (1.11 - 1.87) | 0.00685 |
| *Sociodemographic risk factors* |  |  |
| Age | 1.02 (1.01 - 1.02) | 0.00000 |
| Sex |  |  |
| Female | 1.18 (1.00 - 1.40) | 0.05318 |
| Male | Reference |  |
| Race/Ethnicity |  |  |
| Hispanic | 1.18 (0.91 - 1.52) | 0.20232 |
| NH White | Reference |  |
| NH Black | 1.00 (0.80 - 1.26) | 0.96837 |
| Another NH race and ethnicity | 1.19 (0.80 - 1.76) | 0.38523 |
| Annual household income |  |  |
| $50,000 or more | 0.64 (0.51 - 0.80) | 0.00012 |
| Less than $50,000 | Reference |  |
| Health insurance status |  |  |
| Covered | 0.82 (0.66 - 1.03) | 0.08569 |
| Not covered | Reference |  |
| *History of tobacco use* |  |  |
| Time-varying ENDS use |  |  |
| Non-established ENDS use | Reference |  |
| Former ENDS use | 1.05 (0.90 - 1.24) | 0.51467 |
| Current ENDS use | 1.19 (1.02 - 1.41) | 0.03235 |
| Log of baseline cigarette pack-years / 10 | 1.13 (0.98 - 1.31) | 0.08681 |
| *Other risk factors* |  |  |
| Time-varying secondhand smoking exposure - mean number of 12 hours of exposure in past 7 days | 1.02 (1.00 - 1.05) | 0.07369 |
| Obesity (BMI ≥ 30.0) at baseline |  |  |
| Yes | 1.08 (0.92 - 1.27) | 0.31695 |
| No | Reference |  |
| Asthma exacerbation at baseline |  |  |
| Yes | 2.40 (2.09 - 2.75) | 0.00000 |
| No | Reference |  |
| Abbreviations: GEE, Generalized Estimating Equation; PATH, Population Assessment of Tobacco and Health; IRR, Incidence Rate Ratio; CI, Confidence Interval; NH, non-Hispanic; ENDS, Electronic Nicotine Delivery Systems; BMI, Body Mass Index | | |
| ^a^ Total number of participants = 2883, Total number of observations = 9514 | | |
| ^b^ Adjusting for cigarette and cigar use, sociodemographics, tobacco use history, and other risk factors using Wave 2 weights | | |

| **Table S5**. GEE model predicting incidence rate ratio of asthma exacerbation using Wave 5 weights, PATH Study (Wave 1-5, 2013-19)^a,b^ | | |
| --- | --- | --- |
|  | IRR (95% CI) | p-value |
| Period |  |  |
| 1 (Wave 1 – Wave 2) | Reference |  |
| 2 (Wave 2 – Wave 3) | 1.44 (1.21 - 1.71) | 0.00006 |
| 3 (Wave 3 – Wave 4) | 1.45 (1.23 - 1.70) | 0.00002 |
| 4 (Wave 4 – Wave 5) | 1.19 (0.99 - 1.44) | 0.06415 |
| Time-varying cigarette and cigar use |  |  |
| Non-established cigarette and cigar use | Reference |  |
| Former cigarette or cigar use | 1.05 (0.82 - 1.34) | 0.69994 |
| Exclusive cigarette use | 1.28 (1.02 - 1.60) | 0.03632 |
| Exclusive cigar use | 0.64 (0.36 - 1.15) | 0.13640 |
| Dual use | 1.48 (1.08 - 2.01) | 0.01372 |
| *Sociodemographic risk factors* |  |  |
| Age | 1.01 (1.01 - 1.02) | 0.00000 |
| Sex |  |  |
| Female | 1.26 (1.01 - 1.56) | 0.03901 |
| Male | Reference |  |
| Race/Ethnicity |  |  |
| Hispanic | 1.35 (1.07 - 1.71) | 0.01229 |
| NH White | Reference |  |
| NH Black | 1.02 (0.79 - 1.31) | 0.90855 |
| Another NH race and ethnicity | 1.16 (0.79 - 1.70) | 0.44288 |
| Annual household income |  |  |
| $50,000 or more | 0.63 (0.50 - 0.81) | 0.00035 |
| Less than $50,000 | Reference |  |
| Health insurance status |  |  |
| Covered | 0.84 (0.66 - 1.07) | 0.15394 |
| Not covered | Reference |  |
| *History of tobacco use* |  |  |
| Time-varying ENDS use |  |  |
| Non-established ENDS use | Reference |  |
| Former ENDS use | 1.07 (0.90 - 1.28) | 0.43402 |
| Current ENDS use | 1.21 (0.99 - 1.49) | 0.06788 |
| Log of baseline cigarette pack-years / 10 | 1.16 (0.97 - 1.38) | 0.10896 |
| *Other risk factors* |  |  |
| Time-varying secondhand smoking exposure - mean number of 12 hours of exposure in past 7 days | 1.02 (0.99 - 1.04) | 0.17906 |
| Obesity (BMI ≥ 30.0) at baseline |  |  |
| Yes | 1.05 (0.88 - 1.25) | 0.59540 |
| No | Reference |  |
| Asthma exacerbation at baseline |  |  |
| Yes | 2.41 (2.09 - 2.79) | 0.00000 |
| No | Reference |  |
| Abbreviations: GEE, Generalized Estimating Equation; PATH, Population Assessment of Tobacco and Health; IRR, Incidence Rate Ratio; CI, Confidence Interval; NH, non-Hispanic; ENDS, Electronic Nicotine Delivery Systems; BMI, Body Mass Index | | |
| ^a^ Total number of participants = 2115, Total number of observations = 8047 | | |
| ^b^ Adjusting for cigarette and cigar use, sociodemographics, tobacco use history, and other risk factors using Wave 5 weights | | |

| **Table S6.** GEE model predicting incidence rate ratio of asthma exacerbation excluding respondents diagnosed with chronic obstructive pulmonary disease at baseline, PATH Study (Wave 1-5, 2013-19)^a,b^ | | |
| --- | --- | --- |
|  | IRR (95% CI) | p-value |
| Period |  |  |
| 1 (Wave 1 – Wave 2) | Reference |  |
| 2 (Wave 2 – Wave 3) | 1.50 (1.24 - 1.82) | 0.00006 |
| 3 (Wave 3 – Wave 4) | 1.45 (1.20 - 1.77) | 0.00025 |
| 4 (Wave 4 – Wave 5) | 1.25 (1.03 - 1.52) | 0.02487 |
| Time-varying cigarette and cigar use |  |  |
| Non-established cigarette and cigar use | Reference |  |
| Former cigarette or cigar use | 1.10 (0.87 - 1.39) | 0.42816 |
| Exclusive cigarette use | 1.29 (1.07 - 1.56) | 0.00900 |
| Exclusive cigar use | 0.69 (0.38 - 1.29) | 0.24312 |
| Dual use | 1.64 (1.24 - 2.16) | 0.00061 |
| *Sociodemographic risk factors* |  |  |
| Age | 1.01 (1.01 - 1.02) | 0.00000 |
| Sex |  |  |
| Female | 1.36 (1.11 - 1.67) | 0.00376 |
| Male | Reference |  |
| Race/Ethnicity |  |  |
| Hispanic | 1.29 (1.01 - 1.65) | 0.04428 |
| NH White | Reference |  |
| NH Black | 1.09 (0.86 - 1.39) | 0.46987 |
| Another NH race and ethnicity | 1.25 (0.87 - 1.80) | 0.22990 |
| Annual household income |  |  |
| $50,000 or more | 0.65 (0.52 - 0.83) | 0.00052 |
| Less than $50,000 | Reference |  |
| Health insurance status |  |  |
| Covered | 0.78 (0.60 - 0.99) | 0.04421 |
| Not covered | Reference |  |
| *History of tobacco use* |  |  |
| Time-varying ENDS use |  |  |
| Non-established ENDS use | Reference |  |
| Former ENDS use | 0.98 (0.79 - 1.22) | 0.88785 |
| Current ENDS use | 1.14 (0.92 - 1.41) | 0.22900 |
| Log of baseline cigarette pack-years / 10 | 1.05 (0.89 - 1.25) | 0.53375 |
| *Other risk factors* |  |  |
| Time-varying secondhand smoking exposure - mean number of 12 hours of exposure in past 7 days | 1.02 (0.99 - 1.05) | 0.29428 |
| Obesity (BMI ≥ 30.0) at baseline |  |  |
| Yes | 1.07 (0.90 - 1.27) | 0.46753 |
| No | Reference |  |
| Asthma exacerbation at baseline |  |  |
| Yes | 2.51 (2.13 - 2.95) | 0.00000 |
| No | Reference |  |
| Abbreviations: GEE, Generalized Estimating Equation; PATH, Population Assessment of Tobacco and Health; IRR, Incidence Rate Ratio; CI, Confidence Interval; NH, non-Hispanic; ENDS, Electronic Nicotine Delivery Systems; BMI, Body Mass Index | | |
| ^a^ Total number of participants = 2616, Total number of observations = 8656 | | |
| ^b^ Adjusting for cigarette and cigar use, sociodemographics, tobacco use history, and other risk factors using Wave 1 weights | | |

| **Table S7.** GEE model predicting incidence rate ratio of asthma exacerbation excluding respondents diagnosed with chronic obstructive pulmonary disease, chronic bronchitis, emphysema, or some other lung or respiratory condition at baseline, PATH Study (Wave 1-5, 2013-19)^a,b^ | | |
| --- | --- | --- |
|  | IRR (95% CI) | p-value |
| Period |  |  |
| 1 (Wave 1 – Wave 2) | Reference |  |
| 2 (Wave 2 – Wave 3) | 1.59 (1.29 - 1.98) | 0.00004 |
| 3 (Wave 3 – Wave 4) | 1.53 (1.25 - 1.88) | 0.00006 |
| 4 (Wave 4 – Wave 5) | 1.41 (1.13 - 1.75) | 0.00306 |
| Time-varying cigarette and cigar use |  |  |
| Non-established cigarette and cigar use | Reference |  |
| Former cigarette or cigar use | 1.10 (0.84 - 1.43) | 0.48599 |
| Exclusive cigarette use | 1.36 (1.08 - 1.70) | 0.00848 |
| Exclusive cigar use | 0.71 (0.35 - 1.41) | 0.32200 |
| Dual use | 1.65 (1.24 - 2.20) | 0.00071 |
| *Sociodemographic risk factors* |  |  |
| Age | 1.01 (1.01 - 1.02) | 0.00017 |
| Sex |  |  |
| Female | 1.29 (1.04 - 1.61) | 0.02161 |
| Male | Reference |  |
| Race/Ethnicity |  |  |
| Hispanic | 1.29 (0.98 - 1.70) | 0.06815 |
| NH White | Reference |  |
| NH Black | 1.11 (0.85 - 1.45) | 0.44587 |
| Another NH race and ethnicity | 1.32 (0.86 - 2.04) | 0.20026 |
| Annual household income |  |  |
| $50,000 or more | 0.62 (0.48 - 0.80) | 0.00042 |
| Less than $50,000 | Reference |  |
| Health insurance status |  |  |
| Covered | 0.73 (0.57 - 0.95) | 0.01908 |
| Not covered | Reference |  |
| *History of tobacco use* |  |  |
| Time-varying ENDS use |  |  |
| Non-established ENDS use | Reference |  |
| Former ENDS use | 0.94 (0.73 - 1.21) | 0.62222 |
| Current ENDS use | 1.06 (0.83 - 1.37) | 0.62124 |
| Log of baseline cigarette pack-years / 10 | 1.04 (0.83 - 1.29) | 0.75443 |
| *Other risk factors* |  |  |
| Time-varying secondhand smoking exposure - mean number of 12 hours of exposure in past 7 days | 0.99 (0.96 - 1.03) | 0.74977 |
| Obesity (BMI ≥ 30.0) at baseline |  |  |
| Yes | 1.13 (0.93 - 1.38) | 0.20009 |
| No | Reference |  |
| Asthma exacerbation at baseline |  |  |
| Yes | 2.80 (2.30 - 3.40) | 0.00000 |
| No | Reference |  |
| Abbreviations: GEE, Generalized Estimating Equation; PATH, Population Assessment of Tobacco and Health; IRR, Incidence Rate Ratio; CI, Confidence Interval; NH, non-Hispanic; ENDS, Electronic Nicotine Delivery Systems; BMI, Body Mass Index | | |
| ^a^ Total number of participants = 2351, Total number of observations = 7794 | | |
| ^b^ Adjusting for cigarette and cigar use, sociodemographics, tobacco use history, and other risk factors using Wave 1 weights | | |

| **Table S8**. GEE model predicting incidence rate ratio of asthma excluding premium cigar use at baseline, PATH Study (Wave 1-5, 2013-19)^a,b^ | | |
| --- | --- | --- |
|  | IRR (95% CI) | p-value |
| Period |  |  |
| 1 (Wave 1 – Wave 2) | Reference |  |
| 2 (Wave 2 – Wave 3) | 1.37 (1.18 - 1.60) | 0.00007 |
| 3 (Wave 3 – Wave 4) | 1.38 (1.19 - 1.61) | 0.00005 |
| 4 (Wave 4 – Wave 5) | 1.16 (0.99 - 1.36) | 0.06487 |
| Time-varying cigarette and cigar use |  |  |
| Non-established cigarette and cigar use | Reference |  |
| Former cigarette or cigar use | 1.01 (0.80 - 1.28) | 0.90568 |
| Exclusive cigarette use | 1.27 (1.03 - 1.55) | 0.02438 |
| Exclusive cigar use | 0.72 (0.42 - 1.23) | 0.22288 |
| Dual use | 1.42 (1.08 - 1.87) | 0.01215 |
| *Sociodemographic risk factors* |  |  |
| Age | 1.02 (1.01 - 1.02) | 0.00000 |
| Sex |  |  |
| Female | 1.18 (1.00 - 1.40) | 0.05335 |
| Male | Reference |  |
| Race/Ethnicity |  |  |
| Hispanic | 1.16 (0.91 - 1.49) | 0.23657 |
| NH White | Reference |  |
| NH Black | 0.99 (0.80 - 1.24) | 0.94825 |
| Another NH race and ethnicity | 1.21 (0.81 - 1.79) | 0.34302 |
| Annual household income |  |  |
| $50,000 or more | 0.62 (0.50 - 0.78) | 0.00006 |
| Less than $50,000 | Reference |  |
| Health insurance status |  |  |
| Covered | 0.82 (0.65 - 1.02) | 0.07977 |
| Not covered | Reference |  |
| *History of tobacco use* |  |  |
| Time-varying ENDS use |  |  |
| Non-established ENDS use | Reference |  |
| Former ENDS use | 1.05 (0.90 - 1.23) | 0.49544 |
| Current ENDS use | 1.18 (1.00 - 1.40) | 0.05255 |
| Log of baseline cigarette pack-years / 10 | 1.13 (0.97 - 1.30) | 0.11169 |
| *Other risk factors* |  |  |
| Time-varying secondhand smoking exposure - mean number of 12 hours of exposure in past 7 days | 1.02 (1.00 - 1.05) | 0.08544 |
| Obesity (BMI ≥ 30.0) at baseline |  |  |
| Yes | 1.11 (0.95 - 1.30) | 0.19878 |
| No | Reference |  |
| Asthma exacerbation at baseline |  |  |
| Yes | 2.39 (2.09 - 2.74) | 0.00000 |
| No | Reference |  |
| Abbreviations: GEE, Generalized Estimating Equation; PATH, Population Assessment of Tobacco and Health; IRR, Incidence Rate Ratio; CI, Confidence Interval; NH, non-Hispanic; ENDS, Electronic Nicotine Delivery Systems; BMI, Body Mass Index | | |
| ^a^ Total number of participants = 2858, Total number of observations = 9433 | | |
| ^b^ Adjusting for cigarette and cigar use, sociodemographics, tobacco use history, and other risk factors using Wave 1 weights | | |

| **Table S9.** GEE model predicting incidence rate ratio of asthma exacerbation adjusting for hookah and pipe tobacco use, PATH Study (Wave 1-5, 2013-19)^a,b^ | | |
| --- | --- | --- |
|  | IRR (95% CI) | p-value |
| Period |  |  |
| 1 (Wave 1 – Wave 2) | Reference |  |
| 2 (Wave 2 – Wave 3) | 1.38 (1.19 - 1.60) | 0.00005 |
| 3 (Wave 3 – Wave 4) | 1.38 (1.19 - 1.61) | 0.00005 |
| 4 (Wave 4 – Wave 5) | 1.16 (0.99 - 1.36) | 0.06718 |
| *Time-varying cigarette and cigar use* |  |  |
| Non-established cigarette and cigar use | Reference |  |
| Former cigarette or cigar use | 1.01 (0.79 - 1.27) | 0.95982 |
| Exclusive cigarette use | 1.26 (1.03 - 1.54) | 0.02344 |
| Exclusive cigar use | 0.70 (0.41 - 1.19) | 0.18721 |
| Dual use | 1.43 (1.09 - 1.88) | 0.00981 |
| *Sociodemographic risk factors* |  |  |
| Age | 1.01 (1.01 - 1.02) | 0.00000 |
| Sex |  |  |
| Female | 1.20 (1.01 - 1.43) | 0.03813 |
| Male | Reference |  |
| Race/Ethnicity |  |  |
| Hispanic | 1.17 (0.91 - 1.50) | 0.21109 |
| NH White | Reference |  |
| NH Black | 1.00 (0.80 - 1.25) | 0.97754 |
| Another NH race and ethnicity | 1.21 (0.81 - 1.80) | 0.35131 |
| Annual household income |  |  |
| $50,000 or more | 0.62 (0.49 - 0.77) | 0.00004 |
| Less than $50,000 | Reference |  |
| Health insurance status |  |  |
| Covered | 0.83 (0.66 - 1.03) | 0.09470 |
| Not covered | Reference |  |
| *History of tobacco use* |  |  |
| Time-varying ENDS use |  |  |
| Non-established ENDS use | Reference |  |
| Former ENDS use | 1.06 (0.91 - 1.24) | 0.42528 |
| Current ENDS use | 1.21 (1.04 - 1.42) | 0.01752 |
| Time-varying hookah use |  |  |
| Non-established hookah use | Reference |  |
| Former hookah use | 0.82 (0.63 - 1.08) | 0.15053 |
| Current hookah use | 0.88 (0.59 - 1.30) | 0.50508 |
| Time-varying pipe tobacco use |  |  |
| Non-established pipe tobacco use | Reference |  |
| Former pipe tobacco use | 1.17 (0.79 - 1.73) | 0.43602 |
| Current pipe tobacco use | 0.97 (0.54 - 1.77) | 0.92803 |
| Log of baseline cigarette pack-years / 10 | 1.12 (0.97 - 1.29) | 0.11179 |
| *Other risk factors* |  |  |
| Time-varying secondhand smoking exposure - mean number of 12 hours of exposure in past 7 days | 1.02 (1.00 - 1.04) | 0.08651 |
| Obesity (BMI ≥ 30.0) at baseline |  |  |
| Yes | 1.10 (0.94 - 1.29) | 0.23016 |
| No | Reference |  |
| Asthma exacerbation at baseline |  |  |
| Yes | 2.39 (2.08 - 2.74) | 0.00000 |
| No | Reference |  |
| Abbreviations: GEE, Generalized Estimating Equation; PATH, Population Assessment of Tobacco and Health; IRR, Incidence Rate Ratio; CI, Confidence Interval; NH, non-Hispanic; ENDS, Electronic Nicotine Delivery Systems; BMI, Body Mass Index | | |
| ^a^ Total number of participants = 2882, Total number of observations = 9510 | | |
| ^b^ Adjusting for cigarette and cigar use, sociodemographics, tobacco use history (including hookah and pipe tobacco), and other risk factors using Wave 1 weights | | |

| **Table S10.** GEE model predicting incidence rate ratio of asthma exacerbation adjusting for time-varying annual household income, time-varying health insurance status, and time-varying obesity, PATH Study (Wave 1-5, 2013-19)^a,b^ | | |
| --- | --- | --- |
|  | IRR (95% CI) | p-value |
| Period |  |  |
| 1 (Wave 1 – Wave 2) | Reference |  |
| 2 (Wave 2 – Wave 3) | 1.40 (1.20 - 1.62) | 0.00003 |
| 3 (Wave 3 – Wave 4) | 1.40 (1.21 - 1.63) | 0.00002 |
| 4 (Wave 4 – Wave 5) | 1.18 (1.01 - 1.39) | 0.03982 |
| Time-varying cigarette and cigar use |  |  |
| Non-established cigarette and cigar use | Reference |  |
| Former cigarette or cigar use | 1.01 (0.79 - 1.28) | 0.95169 |
| Exclusive cigarette use | 1.27 (1.03 - 1.57) | 0.02496 |
| Exclusive cigar use | 0.71 (0.42 - 1.20) | 0.20001 |
| Dual use | 1.42 (1.06 - 1.90) | 0.01796 |
| *Sociodemographic risk factors* |  |  |
| Age | 1.01 (1.01 - 1.02) | 0.00000 |
| Sex |  |  |
| Female | 1.20 (1.01 - 1.42) | 0.03335 |
| Male | Reference |  |
| Race/Ethnicity |  |  |
| Hispanic | 1.17 (0.90 - 1.52) | 0.23995 |
| NH White | Reference |  |
| NH Black | 1.03 (0.82 - 1.29) | 0.80068 |
| Another NH race and ethnicity | 1.27 (0.87 - 1.84) | 0.20941 |
| Time-varying annual household income |  |  |
| $50,000 or more | 0.71 (0.58 - 0.86) | 0.00076 |
| Less than $50,000 | Reference |  |
| Time-varying health insurance status |  |  |
| Covered | 0.88 (0.76 - 1.02) | 0.08817 |
| Not covered | Reference |  |
| *History of tobacco use* |  |  |
| Time-varying ENDS use |  |  |
| Non-established ENDS use | Reference |  |
| Former ENDS use | 1.06 (0.91 - 1.24) | 0.46970 |
| Current ENDS use | 1.20 (1.03 - 1.41) | 0.02214 |
| Log of baseline cigarette pack-years / 10 | 1.15 (0.99 - 1.33) | 0.06002 |
| *Other risk factors* |  |  |
| Time-varying secondhand smoking exposure - mean number of 12 hours of exposure in past 7 days | 1.02 (1.00 - 1.05) | 0.04203 |
| Time-varying obesity (BMI ≥ 30.0) |  |  |
| Yes | 1.08 (0.95 - 1.23) | 0.25394 |
| No | Reference |  |
| Asthma exacerbation at baseline |  |  |
| Yes | 2.39 (2.08 - 2.74) | 0.00000 |
| No | Reference |  |
| Abbreviations: GEE, Generalized Estimating Equation; PATH, Population Assessment of Tobacco and Health; IRR, Incidence Rate Ratio; CI, Confidence Interval; NH, non-Hispanic; ENDS, Electronic Nicotine Delivery Systems; BMI, Body Mass Index | | |
| ^a^ Total number of participants = 2883, Total number of observations =9262 | | |
| ^b^ Adjusting for cigarette and cigar use, sociodemographics, tobacco use history, and other risk factors using Wave 1 weights | | |

| **Table S11**. GEE model predicting incidence rate ratio of asthma exacerbation using disaggregated exposure variable, PATH Study (Wave 1-5, 2013-19)^a,b^ | | |
| --- | --- | --- |
|  | IRR (95% CI) | p-value |
| Period |  |  |
| 1 (Wave 1 – Wave 2) | Reference |  |
| 2 (Wave 2 – Wave 3) | 1.38 (1.19 - 1.60) | 0.00005 |
| 3 (Wave 3 – Wave 4) | 1.39 (1.19 - 1.61) | 0.00004 |
| 4 (Wave 4 – Wave 5) | 1.16 (0.99 - 1.36) | 0.06099 |
| Time-varying cigarette and cigar use |  |  |
| Never use | Reference |  |
| Former experimental cigarette or cigar use | 1.06 (0.80 - 1.39) | 0.69219 |
| Current experimental cigarette or cigar use | 1.18 (0.83 - 1.68) | 0.34493 |
| Former cigarette or cigar use | 1.05 (0.79 - 1.41) | 0.73370 |
| Exclusive cigarette use | 1.31 (1.02 - 1.69) | 0.03662 |
| Exclusive cigar use | 0.73 (0.43 - 1.23) | 0.23532 |
| Dual use | 1.47 (1.05 - 2.05) | 0.02384 |
| *Sociodemographic risk factors* |  |  |
| Age | 1.02 (1.01 - 1.02) | 0.00000 |
| Sex |  |  |
| Female | 1.19 (1.01 - 1.41) | 0.04260 |
| Male | Reference |  |
| Race/Ethnicity |  |  |
| Hispanic | 1.17 (0.91 - 1.49) | 0.21081 |
| NH White | Reference |  |
| NH Black | 1.00 (0.80 - 1.24) | 0.96856 |
| Another NH race and ethnicity | 1.21 (0.82 - 1.78) | 0.32859 |
| Annual household income |  |  |
| $50,000 or more | 0.62 (0.49 - 0.78) | 0.00007 |
| Less than $50,000 | Reference |  |
| Health insurance status |  |  |
| Covered | 0.82 (0.66 - 1.03) | 0.09061 |
| Not covered | Reference |  |
| *History of tobacco use* |  |  |
| Time-varying ENDS use |  |  |
| Non-established ENDS use | Reference |  |
| Former ENDS use | 1.05 (0.90 - 1.22) | 0.53518 |
| Current ENDS use | 1.19 (1.02 - 1.39) | 0.02818 |
| Log of baseline cigarette pack-years / 10 | 1.12 (0.97 - 1.30) | 0.12022 |
| *Other risk factors* |  |  |
| Time-varying secondhand smoking exposure - mean number of 12 hours of exposure in past 7 days | 1.02 (1.00 - 1.05) | 0.07955 |
| Obesity (BMI ≥ 30.0) at baseline |  |  |
| Yes | 1.11 (0.94 - 1.30) | 0.20656 |
| No | Reference |  |
| Asthma exacerbation at baseline |  |  |
| Yes | 2.40 (2.10 - 2.75) | 0.00000 |
| No | Reference |  |
| Abbreviations: GEE, Generalized Estimating Equation; PATH, Population Assessment of Tobacco and Health; IRR, Incidence Rate Ratio; CI, Confidence Interval; NH, non-Hispanic; ENDS, Electronic Nicotine Delivery Systems; BMI, Body Mass Index | | |
| ^a^ Total number of participants = 2883, Total number of observations = 9514 | | |
| ^b^ Adjusting for cigarette and cigar use, sociodemographics, tobacco use history, and other risk factors using Wave 1 weights | | |
